# Supplementary material for: Collaborative intelligence in AI: Evaluating the performance of a council of AIs on the USMLE
Source: PLOS Digit Health. 2025 Oct 9;4(10):e0000787. doi: 10.1371/journal.pdig.0000787 (PMC12510544; doi:10.1371/journal.pdig.0000787)
Supplement: S2 File — (ZIP) [file pdig.0000787.s002.zip › S2 Data Raw data files containing unprocessed question inputs and LLM outputs including transcripts of deliberations/STEP3_Question-136-1_Type-MC-NJ.docx]

**Facilitator** **[Round 1]:** A 30-year-old man comes to the office because of an itchy and painful facial rash that has worsened since he first noticed it 1 day ago. He has no other symptoms and says he otherwise has been generally healthy. Medical history is unremarkable and he takes no medications. He does not use any substances. He is unmarried and is not currently in a romantic relationship but has been sexually active with multiple female partners during the past year. Vital signs are within normal limits. Physical examination shows an erythematous macular rash with several 1- to 2-mm raised vesicles that do not cross the midline over the left forehead and periorbital area. There are no other abnormalities. Acyclovir therapy is initiated. Which of the following is the most appropriate next step? (A) Referral to a dermatologist (B) Serum HIV antibody test (C) Skin biopsy (D) Tzanck test

**AI_B:** (1) RESTATING THE QUESTION: The scenario presents a case of a 30-year-old male who is reporting an itchy and painful facial rash that showed worsening over one day. The vesicular rash, which is red and does not cross the midline of his face, is the most notable presentation. His history indicates he's otherwise healthy, but has had multiple sexual partners. Acyclovir, an antiviral medication, was initiated and we're asked to determine the best subsequent step. (2) PLANNING: The presented symptoms and the initiation of acyclovir suggest a possible herpes simplex virus (HSV) infection. Alternatives for the cause of the rash include other infections, contact dermatitis, or autoimmune conditions. Considering the rash pattern and given acyclovir was initiated, we'll lean on the herpes infection assumption. The possible options pertain to diagnosis confirmation (Tzanck test or skin biopsy), HIV testing (as there is a known correlation between HSV and HIV), or specialist referral. (3) CLINICAL REASONING: Starting from the assumption of a herpes infection, our efforts should be focused on confirming this diagnosis, as well as evaluating potential complications or associated conditions. Beginning acyclovir treatment indicates suspicion of herpes virus infection, most likely herpes simplex virus type 1 (HSV-1), given the location of the rash (Herpes Zoster, another condition that could manifest through a unilateral vesicular rash, tends to occur in older, immunosuppressed patients). Therefore, it would be beneficial to confirm the diagnosis using a Tzanck test (D), a smear of an opened skin vesicle to detect multinucleated giant cells, commonly used in diagnosing HSV-1. (4) CONCLUDING: Upon considering this information, it appears the best course of action would be proceeding with diagnostic confirmation before referring him to a specialist or conducting further tests. The Tzanck test (D), being a simple and quick test, seems to be the appropriate next step as its result can provide more information necessary for the patient's case. (5) RESPONSE: Therefore, the most appropriate next step for this patient should be a Tzanck test (D).

**AI_A:** (1) RESTATING THE QUESTION: We are presented with a case of a 30-year-old man who has appeared at the physician's office with a rapidly worsening itchy, painful facial rash. His vital signs are normal and there are no other symptoms or health issues reported. He has been sexually active with various partners. Upon examination, it is observed that he has an erythematous macular rash with small vesicles over the left side of his forehead and around his eye, which doesn't extend across the midline of his face. Acyclovir therapy was initiated immediately, based on these findings. We need to determine the most appropriate next step from the options provided: referral to a dermatologist, serum HIV antibody test, skin biopsy, or Tzanck test. (2) PLANNING: Let's consider the assumptions. The fact that Acyclovir, an antiviral medication, was initiated suggests an assumption of a viral infection, most likely Herpes simplex virus type 1 (HSV-1), based on the rash description and location. An alternate assumption could be that the rash is caused by another type of skin condition requiring different tests or referral for specialized care. Let's evaluate these assumptions. (3) CLINICAL REASONING: This patient's unilaterally distributed vesicles and the location around the forehead and periorbital area suggest herpes zoster ophthalmicus, not HSV-1. Herpes zoster ophthalmicus is a reactivation of the varicella-zoster virus. Acyclovir is a typical treatment for zoster and herpes infections, due to its inhibitory action on viral DNA replication. The next most important step would logically be to confirm the diagnosis. The Tzanck test is used for this and identifies herpes virus-infected cells in cutaneous lesions caused by HSV-1, HSV-2, or Varicella-Zoster Virus (VZV). Although the patient may require dermatological or HIV assessment later, given his sexual history, the immediate priority should be confirming the suspected viral infection to guide and justify the initiated treatment. (4) CONCLUDING: The next step in management, after the initiation of Acyclovir, should be a test that confirms the initial clinical suspicion of a herpes zoster ophthalmicus infection. The Tzanck test identifies herpes virus-infected cells in skin lesions. Although dermatological or HIV evaluation may be necessary in future, at this point immediate confirmation of the infection is of greater significance. (5) RESPONSE: The correct answer is (D) Tzanck test.

**AI_E:** 1) RESTATING THE QUESTION: A 30-year-old male patient arrived at the clinic presenting with an itchy and painful facial rash that has worsened over the last day. His medical history doesn't indicate any pre-existing conditions and he isn't on any medication. He leads an active sexual life with multiple partners but does not use any substances. His vital signs are normal. The physical examination reveals a reddish, flat rash with several small bumps that are restricted to one side of the forehead and around the eye. Based on these findings, the initial therapy with Acyclovir was started. Now we need to decide the next appropriate step. 2) PLANNING: The patient's clinical presentation can be suggestive of a viral infection which would explain why Acyclovir, an antiviral medication, has been initiated. The pattern of the rash, its quick appearance, and the fact that it doesn't cross the midline suggest herpes simplex virus (HSV-1) or herpes zoster virus (HZV) infections, both of which could cause such symptoms. If it were HSV, the patient's active sexual life might be relevant, and the chosen therapy is correct as Acyclovir is used to treat both HSV and HZV infections. However, to confirm which virus is responsible or to rule out any other skin conditions, further testing might be needed. 3) CLINICAL REASONING: Given the symptoms, the patient is most likely suffering from an acute viral infection. Referral to a dermatologist (option A) is not the priority since this is a manageable primary care scenario with an apparent herpes infection. A serum HIV test (option B) could be considered later given the patient's sexual history, yet there's no immediate suspicion for HIV given the absence of other symptoms, history, or risk factors apart from multiple sexual partners. Skin biopsy (option C) isn't the most immediate next step since it's invasive, and the clinical presentation suggests a diagnosis that can be assessed through other less invasive means. 4) CONCLUDING: Hence, the primary approach in this case includes confirming the initial diagnosis of a herpes infection which can be done through laboratory tests, such as Tzanck smear, which is useful for identifying cells with viral changes. Conclusively, this test is a quick and less invasive method to provide diagnostic evidence for HSV or HZV. 5) RESPONSE: Therefore, the most appropriate next step in this case would be a 'Tzanck test' (option D).

**AI_C:** Restating the question: The clinical presentation here suggests that a 30-year-old man is suffering from an uncomfortable, irritating facial rash that's become notably worse over the last day. Apart from this, he says he feels well and his past medical history returns no significant results. His lifestyle is sexually active with multiple partners but he is not currently engaged in a relationship. Clinical examination reveals an erythematous (reddish) macular (flat) rash with a number of raised vesicles (blister-like eruptions) on his left forehead and around his eyes, which don't cross the midline. Other than this, there are no notable abnormalities. His primary care practitioner has started him on acyclovir. The next step in his treatment must be determined from the following options: referral to a dermatologist, a serum HIV antibody test, a skin biopsy, or a Tzanck test. Planning: Considering the presented symptoms and physical examination results, the man's condition closely resembles herpes zoster, especially noting the unilateral vesicular rash. As herpes zoster is a viral infection primarily handled with antiviral treatment (in this case, acyclovir), initiation of this therapy suggests the working diagnosis. The Tzanck test (option D) may be performed as a quick diagnostic tool to confirm presence of herpes virus, revealing multi-nucleated giant cells. Other assumptions such as dermatological conditions needing specialist referral (option A), possible HIV infection (option B), or requiring a more invasive skin biopsy (option C) may be less prioritised clinically. Clinical reasoning: Herpes zoster, commonly known as shingles, is a condition that is primarily diagnosed based on the clinical picture. It is characterized by a painful, blistering rash usually confined to one dermatome and does not cross the midline, as is seen in this patient. Acyclovir is a known antiviral medication used in the treatment of infections caused by herpes viruses, including herpes zoster. The initiation of acyclovir therapy indicates that the treating physician suspected a herpes virus infection, like herpes zoster. This is consistent with the patient's presentation. The Tzanck test, although less commonly used due to the availability of more accurate diagnostic methods such as polymerase chain reaction, can still be used as a quicker, available, less expensive test to identify herpes virus infections, by showing multi-nucleated giant cells on microscopic exam. Concluding: Given the patient's clinical presentation, the presence of an itchy, painful facial rash that is confined to one dermatome, does not cross the midline, and the initiation of acyclovir therapy suggest a diagnosis of herpes zoster. The Tzanck test, despite some limitations, can be useful in this context as a quick and relatively inexpensive way to confirm the diagnosis. Response: Therefore, the most appropriate next step would be to perform a Tzanck test (D). This diagnostic resource, while a bit outdated, can provide quick results to confirm herpes virus infections, which aligns with the patient's presenting symptoms and initial treatment approach. A full diagnostic confirmation would allow for a more guided treatment strategy to hopefully alleviate the patient's discomfort.

**AI_D:** 1) RESTATING THE QUESTION: The query involves a 30-year-old man who presents with an itchy and painful rash on his face that has been worsening over a day. His medical and drug history is uncomplicated and he's been fairly healthy so far. The rash is a red, flat rash characterized by raised blisters around 1-2 mm in size, which does not cross the midline on the left forehead and the region around his eyes. Consequently, he has been prescribed acyclovir therapy. We are required to figure out the best course of action moving forward. Acyclovir is an antiviral drug commonly utilized to handle conditions triggered by the herpes simplex viruses HSV-1 (typically causing oral herpes) and HSV-2 (usually causing genital herpes). Hence, the assumption here may be that the patient's condition is due to herpes simplex virus. 2) PLANNING: The presence of a rash not crossing the midline and the acyclovir prescription suggest a probable cause of Herpes simplex virus specifically Herpes Zoster which has a predilection in showing a pattern of not crossing the midline. However, could there possibly be other causes for this rash? If not, how can we confirm the cause to be indeed Herpes Zoster especially given his sexual history? 3) CLINICAL REASONING: In herpes zoster infection (shingles), lesions often maintain a unilateral distribution, strictly confined to a single dermatome, which correlates with the findings in this patient. Shingles is caused by the reactivation of the varicella-zoster virus (herpes zoster), the same virus responsible for chickenpox. Acyclovir is a suitable treatment, as it's an antiviral agent effective against herpesviruses. While a dermatology referral (A) or a skin biopsy (C) may be helpful in other circumstances, considering the classic clinical presentation and the fact that the patient is already on a therapeutically effective antiviral regimen, they may not be the most appropriate next steps. However, given that herpes zoster can resurface in times of stress on the immune system, including HIV infection, conducting an HIV test (Option B) might be considered prudent given his history of multiple sexual partners. Although HIV tests are generally advised for patients with recurrent or atypical herpes infections more correlated with HSV-2, not herpes zoster. Option D, the Tzanck test, which involves staining scraped cells from vesicles to identify intranuclear inclusions (multinucleated giant cells), can be used to confirm a suspected diagnosis of HSV or VZV, but it doesn't differentiate between the two. 4) CONCLUDING: Given that his symptoms are congruent with the presentation of Herpes Zoster and the first line of treatment has already been initialized, it would make the most sense to conduct a test that can confirm this diagnosis. 5) RESPONSE: Hence, the most appropriate next step is (D) Tzanck test to confirm the provisional diagnosis of herpes zoster.

**Facilitator** **[Round 2]:** Summary: All council members agree that the patient's symptoms suggest a herpes virus infection, likely herpes zoster ophthalmicus, given the unilateral distribution of the rash and the initiation of acyclovir therapy. They all conclude that the Tzanck test, which identifies herpes virus-infected cells in skin lesions, is the most appropriate next step to confirm the diagnosis. However, there are slight differences in their reasoning. AI_A and AI_C emphasize the need to confirm the diagnosis to justify the initiated treatment. AI_B and AI_E highlight the Tzanck test's simplicity and speed. AI_D, while agreeing with the others, also mentions the possibility of an HIV test due to the patient's sexual history, but ultimately agrees that the Tzanck test is the most immediate next step. Key Differences: The key differences in the council members' perspectives lie in their reasoning for choosing the Tzanck test and their consideration of other potential tests or referrals. AI_A and AI_C focus on the need to confirm the diagnosis to justify the treatment, while AI_B and AI_E emphasize the Tzanck test's simplicity and speed. AI_D uniquely brings up the possibility of an HIV test due to the patient's sexual history, but agrees that the Tzanck test is the most immediate next step. Question for Council Members: While all council members agree on the Tzanck test as the next step, there is a slight difference in the reasoning. Could you elaborate on why you believe the Tzanck test is the most appropriate next step, and under what circumstances might you consider other tests or referrals, such as an HIV test or dermatologist referral?. Based on your preceding response, how would you answer the original question: [A 30-year-old man comes to the office because of an itchy and painful facial rash that has worsened since he first noticed it 1 day ago. He has no other symptoms and says he otherwise has been generally healthy. Medical history is unremarkable and he takes no medications. He does not use any substances. He is unmarried and is not currently in a romantic relationship but has been sexually active with multiple female partners during the past year. Vital signs are within normal limits. Physical examination shows an erythematous macular rash with several 1- to 2-mm raised vesicles that do not cross the midline over the left forehead and periorbital area. There are no other abnormalities. Acyclovir therapy is initiated. Which of the following is the most appropriate next step? (A) Referral to a dermatologist (B) Serum HIV antibody test (C) Skin biopsy (D) Tzanck test]

**AI_B:** (1) RESTATING THE QUESTION: The query is about determining the appropriate next step in investigating a 30-year-old man's condition. The man has a painful facial rash that's limited to his left forehead and periorbital region and has been prescribed acyclovir, a medication often used for viral infections caused by herpes viruses. Our choices include referring to a dermatologist for further examination, conducting a serum HIV antibody test, obtaining a skin biopsy, or carrying out a Tzanck test. The Tzanck test is a rapid diagnosis tool used to detect cells infected with herpes viruses. (2) PLANNING: The assumptions are that this person's diagnosis is clear, that acyclovir was appropriately prescribed, and that no other tests are necessary. However, an alternate assumption might be that the diagnosis needs to be solidified with further testing. This could provide relevant information to support the use of acyclovir or help clinicians prepare for other potential disease complications. Questioning this assumption leads me to prefer the alternative; I would give priority to confirming the diagnosis. (3) CLINICAL REASONING: The fact that acyclovir has been initiated is a significant point. If the physician believes the patient to have a herpes virus infection, specifically Herpes Zoster (also known as shingles), this would explain the acyclovir prescription. Given the characteristic vesicular rash that doesn't cross the midline, the lack of other symptoms, the rash's expeditious development, and the patient’s age, a herpes zoster ophthalmicus diagnosis is likely. The itchy, painful blistering rash, limited distribution, and rapid development would all support this. If this is the case, using an on-the-spot diagnostic test like the Tzanck test for visual confirmation of herpes virus-infected cells would be beneficial as it is simple, quick, and can provide immediate support for the diagnosis. (4) CONCLUDING: So, while the rash's characteristics already suggest a herpes virus infection and the physician has initiated acyclovir, corroborating this with a Tzanck test would further support the herpes zoster ophthalmicus diagnosis, reinforce the acyclovir therapy, and could help anticipate potential complications such as eye involvement and post-herpetic neuralgia. (5) RESPONSE: Based on the presented information and the step-by-step clinical thought process used above, the most suitable next step would be to perform a Tzanck test (D).

**AI_D:** RESTATING THE QUESTION: The question presents a case of a 30-year-old man with an itchy and painful rash on his face, who otherwise reports being generally healthy. The rash presents as an erythematous macular with small vesicles on the left side of his forehead and around the eyes, not crossing the midline. Acyclovir treatment has been initiated, which is typically used for viral infections such as herpes simplex and herpes zoster. The task here is to determine the most appropriate step following the initiation of acyclovir. PLANNING: The options include a dermatologist referral, an HIV antibody test, a skin biopsy, and a Tzanck test. In generating alternate assumptions, we need to consider why acyclovir treatment was started. Given the rash's presentation, it is highly likely to be due to herpes zoster. The Tzanck test is a quick and easy method to diagnose herpes virus infections. An HIV test might be suggested due to the patient's sexual history, but there is no other symptom or sign that implies immunosuppression. A skin biopsy can diagnose various skin diseases, but it's more invasive and less specific for herpes infections. Finally, a dermatologist referral might be considered if the diagnosis was unclear, but given the patient's symptoms and acyclovir initiation, herpes zoster seems the probable cause. CLINICAL REASONING: Whereas other diagnostic tests might provide additional information or confirmatory results, the Tzanck test is most directly related to the assumed diagnosis behind the choice of acyclovir treatment. A referral to a dermatologist or a more invasive biopsy is not warranted at least until after the results of the much simpler Tzanck test have been obtained. As for an HIV test, it could be potentially important considering the patient's history of multiple sexual partners, but there is no explicit reason based on symptoms to assume HIV infection or any other cause of immunosuppression. CONCLUDING: Considering the patient's symptoms, the likely diagnosis, the initiated acyclovir treatment, and the options available, I would say that the most clinically appropriate action would be to conduct a Tzanck test to confirm the suspicion of a herpes zoster infection. The test is simple, quick, and its results will directly confirm or refute the assumed condition which the treatment is already targeting. RESPONSE: Therefore, I agree with the other council members that option (D) Tzanck test is the most appropriate next step in the management of this patient's condition.

**AI_A:** 1) RESTATING THE QUESTION: This query is asking the most appropriate next step to take in the management of a 30-year-old male who presents with an itchy and painful facial rash which seems to align with a possible herpes virus infection on the left side of his forehead and periorbital area, given that acyclovir therapy has been started. The answer choices suggest possible ways to confirm the diagnosis, assess for underlying conditions, or refer to a specialist. 2) PLANNING: The assumptions here are that the diagnosed and treated condition is indeed herpes virus infection and subsequent management should follow this line. But, a different assumption might be to consider the possibility of other associated or underlying conditions (given his sexual history). In this case, the question arises: Should we primarily focus on diagnosing and managing the immediate concern (herpes infection) or take into account other factors that could influence overall health status (such as potential sexually transmitted diseases including HIV)? It is imperative to prioritize the immediate clinical concern - a possible herpes infection. 3) CLINICAL REASONING: Given the symptomatology, a herpes virus infection (probably Herpes Zoster) appears likely. Acyclovir therapy has been started, suggesting initial clinical suspicion is in that direction. While HIV or other STDs could have a role given the patient's sexual history, the lack of other systemic symptoms or signs may not necessarily point towards an acute systemic disease. The clinical presentation is more focal, suggesting a localized condition. Therefore, it seems reasonable, for now, to primarily focus on the direct management of the rash. 4) CONCLUDING: In light of the above, the primary need is to ascertain optimal management of the apparent condition - a probable Herpes Zoster infection. This would involve confirming the diagnosis at the earliest to justify the treatment initiated and then watch for response. It also makes sense to have a low threshold for considering other associated conditions like HIV, but they may not be the immediate priority unless there are specific signs or symptoms. 5) RESPONSE: With respect to the question asked, the most appropriate next step seems to be the Tzanck test (Choice D), as it can effectively confirm herpes virus infection using cells from the skin lesions and this can align the therapeutic actions. Nevertheless, the patient's sexual history should be kept in mind for possible future evaluations. An HIV test or dermatologist referral may indeed be considered, but only if the clinical course, response to treatment, or additional symptoms or signs indicate accordingly.

**AI_E:** RESTATING THE QUESTION: The question asks us to identify the most suitable next step in managing a 30-year-old male patient presenting with a unilateral erythematous macular rash with several vesicles on the left side of his forehead and periorbital area. This patient's rash has been worsening over the past day and is itchy and painful. There are no other health concerns, and the patient's sexual history over the past year includes multiple female partners. Acyclovir therapy has been started, indicating a suspected viral infection, possibly due to a herpesvirus. PLANNING: This patient's symptoms and the noted distribution of the rash on his face suggest an infection caused by the varicella-zoster virus (VZV) known as herpes zoster or shingles. This virus manifests itself along a particular dermatome without crossing the midline. Acyclovir, an antiviral drug, is appropriate for treating herpesvirus infections. An assumption is that the symptoms are caused by herpes zoster and not another skin condition, due to the specific pattern in which it presents. However, alternate assumptions could be other skin conditions, such as contact dermatitis, impetigo, or another sexually transmitted infection considering his sexual history. That makes the most significant assumption to prioritize—the one of herpes zoster infection. CLINICAL REASONING: Herpes zoster presents with a painful, itchy rash that usually occurs in a unilateral dermatomal distribution, fitting this patient's symptoms. The absence of symptoms beyond the skin suggests a localized infection, further pointing towards herpes zoster rather than systemic illnesses. The unilateral nature of the rash, with vesicles, significantly leans towards VZV infection. A Tzanck test can be used to identify herpesvirus-infected cells in skin lesions, providing rapid confirmation. This choice would be more important than running an HIV test at this time because the symptoms presently align with a herpes zoster outbreak, and this can be quickly confirmed. CONCLUDING: The appropriate next step would be to confirm the suspected herpes zoster diagnosis. Given the speed and simplicity of the Tzanck test, it offers the best immediate investigative method for support of the suspected diagnosis. Differentiating herpes zoster from other skin conditions such as impetigo, contact dermatitis, or another sexually transmitted infection through rapid diagnosis can guide management and patient education. RESPONSE: Therefore, the most appropriate next step in the management of this patient would be to conduct a Tzanck test (Option D). This test would allow a quick diagnosis of the herpesvirus infection, supporting the current acyclovir therapy and eliminating the need for other invasive or time-consuming procedures at this time. Should the Tzanck test result not support a herpes zoster diagnosis, other testing, including an HIV test or referral to dermatology may be appropriate.

**AI_C:** (1) RESTATING THE QUESTION: The question asks us what the most appropriate next course of action is for diagnosing a 30-year-old man who has presented at the office with a one-day old itchy and painful facial rash. The rash is erythematous, macular with several small raised vesicles that restrict to one side of his face. He has no known health issues or medication usage, does not consume any substances, and is sexually active with multiple partners. Acyclovir therapy has been initiated to treat his condition. The options provided include referring the patient to a dermatologist, conducting a serum HIV antibody test, doing a skin biopsy or performing a Tzanck test. (2) PLANNING: This case presents with unilateral vesicular lesions on an erythematous base which is localized to the forehead and periorbital region without crossing the midline and the absence of other complaints. These point to the infection being likely Herpes Zoster Ophthalmicus. The administration of Acyclovir attests to this preliminary diagnosis. However, to make sure of it, a Tzanck test should be done. It's a quick, convenient lab test that highlights viral cytopathic changes, indicative of herpetic infections. However, one must bear in mind that it doesn't distinguish between Herpes Simplex and Varicella-Zoster Virus. A skin biopsy is a feasible option, but it is invasive and time-consuming compared to a Tzanck test. As for the referral to a dermatologist or ordering a Serum HIV test, they can be kept as considerations in the overall healthcare plan but are not immediate concerns in this case. (3) CLINICAL REASONING: For a patient suffering from an acute itchy, vesicular rash with no other systemic ailments, attempting a diagnosis is primary. In this case, herpes zoster ophthalmicus is indicated. Acyclovir has already been started which attests to this suspicion, however, as physicians, it is our duty to confirm this diagnosis. A Tzanck test would be the best option since it will confirm the presence of the Herpes virus. The fast and less invasive Tzanck test outweighs the utility of the invasive skin biopsy, which is unnecessary at this stage. The patient's sexual history and the absence of a monogamous relationship calls for HIV screening, but this doesn't need to be the most immediate step. And while a dermatologist may indeed provide valuable insight into the condition, it is not an immediate necessity in this case. (4) CONCLUDING: To conclude, considering the clinical presentation of a unilateral forehead and periorbital vesicular rash, the likely diagnosis is herpes zoster ophthalmicus. Acyclovir therapy is already begun, but to confirm the presence of a herpes virus, we should perform a Tzanck test. It's fast, less invasive, and provides a definite cell analysis to confirm our suspicion. (5) RESPONSE: Thus, the most appropriate next step in the management of this patient's condition is to perform a Tzanck test (D), to confirm the presence of a herpes virus causing the patient's symptoms.

**Facilitator** The consensus among the AI responses is that the most appropriate next step in managing the 30-year-old male patient's condition is to perform a Tzanck test (Option D). This conclusion is based on the patient's presentation of an itchy and painful facial rash, which is erythematous and macular with several small raised vesicles, localized to the left side of his forehead and periorbital area. The rash's characteristics, along with the initiation of acyclovir therapy, suggest a probable herpes virus infection, likely Herpes Zoster Ophthalmicus. The Tzanck test is a quick, convenient, and less invasive diagnostic tool that can effectively confirm a herpes virus infection using cells from the skin lesions, thereby aligning the therapeutic actions. While the patient's sexual history and the absence of a monogamous relationship call for HIV screening, this doesn't need to be the most immediate step. Referral to a dermatologist or a more invasive skin biopsy is not warranted at least until after the results of the Tzanck test have been obtained. Therefore, the Tzanck test is the most clinically appropriate action to confirm the suspicion of a herpes zoster infection.
